# Anserine/Carnosine Supplementation Preserves Blood Flow in the Prefrontal Brain of Elderly People Carrying APOE e4

**DOI:** 10.14336/AD.2017.0809

**Published:** 2018-06-01

**Authors:** Qiong Ding, Kitora Tanigawa, Jun Kaneko, Mamoru Totsuka, Yoshinori Katakura, Etsuko Imabayashi, Hiroshi Matsuda, Tatsuhiro Hisatsune

**Affiliations:** ^1^Department of Integrated Biosciences, Graduate School of Frontier Sciences, and; ^2^Department of Applied Biochemistry, Graduate School of Agriculture and Life Sciences, The University of Tokyo, Tokyo, Japan; ^3^Graduate School of Systems Life Sciences, Kyushu University, Higashi-ku, Fukuoka, Japan; ^4^Integrative Brain Imaging Center (IBIC), National Center of Neurology and Psychiatry, Tokyo, Japan

**Keywords:** Alzheimer’s Disease, ASL, DTI, verbal memory, RCT, APOE e4

## Abstract

In a previously reported double-blind, randomized controlled trial (RCT), we demonstrated that daily supplementation with anserine (750 mg) and carnosine (250 mg) improves brain blood flow and memory function in elderly people. Here, we conducted a sub-analysis of MRI data and test scores from the same RCT to determine whether anserine/carnosine supplementation specifically benefits elderly people carrying the APOE e4 allele, which is a risk gene for accelerated brain aging and for the onset of Alzheimer’s Disease. We collected data from 68 participants aged 65 years or older who received anserine/carnosine supplementation (ACS) or placebo for 12 months. Subjects were assessed at the start and end of the trial using several neuropsychological tests, including the Wechsler Memory Scale-Logical Memory (WMS-LM). We also collected two types of MRI data, arterial spin labeling (ASL) and diffusion tensor imaging (DTI) at the start and end of the trial. We found that ACS significantly preserved verbal memory (WMS-LM, F[1,65] = 4.2003, *p* = 0.0445) and blood flow at frontal areas of the brain (FWE_cluster level_, *p* < 0.001). Sub-analysis based on the APOE4 genotype showed a significant preservation of blood flow (*p* = 0.002, by ASL analysis) and white-matter microstructure (*p* = 0.003, by DTI analysis) at prefrontal areas in APOE4^+^ subjects in the active group, while there was no significant difference between APOE4^-^ subjects in the active and placebo groups. The effect of ACS in preserving brain structure and function in elderly people carrying APOE4 should be verified by further studies.

Alzheimer’s disease (AD) is a chronic neurodegenerative disease that is responsible for 60-70% of dementia [1]. Diagnosis and intervention in the early stages, when symptoms first begin to appear, can reduce the number of individuals with AD. The onset of AD symptoms typically begins with a subtle decline in memory. Lifestyle improvements, such as better nutrition and increased mental and physical activity, [2,3], may slow this process and delay the onset of AD. In previous studies [4,5], we investigated the effect of daily dietary supplements containing anserine (beta-alanyl-3-methyl-L-histidine) and carnosine (beta-alanyl-L-histidine) on the preservation of memory function in healthy elderly people.

Carnosine is an endogenous dipeptide that consists of beta-alanine and histidine and is present in skeletal muscle in the millimolar range and in the vertebrate brain in the hundred-micromolar range [6,7]. High levels of anserine, a natural carnosine derivative, are found in the skeletal breast muscle of chicken. Anserine is equivalent to carnosine in physiological function except that it is not cleaved by human carnosinase [8,9]. Double-blind randomized control trials (RCT) conducted by our group and others have demonstrated that anserine/carnosine supplementation (ACS) preserves verbal memory performance in elderly people [4,5,10]. We assessed verbal episodic memory with the Wechsler Memory Scale (WMS-R LM) [11].

Normal aging is associated with diminished brain perfusion [12]. We previously suggested that ACS may suppress this age-related decline in brain perfusion [5]. The strongest known heritable risk factor for AD is the ε4 allele of apolipoprotein E (APOE) [13-15]; this allele is also a risk factor for an accelerated age-related decline in brain perfusion [16-18].

Here, we assessed whether ACS preserved brain blood flow in healthy elderly subjects carrying the APOE e4 allele. We also examined white-matter abnormalities by diffusion MRI, on which decreased blood flow at the white matter causes abnormal hyperintensity of the white-matter [19,20] and decreased fraction anisotropy values [21,22]. We compared psychological test scores and MRI data between elderly subjects in the ACS-treated (active) and placebo groups according to the presence or absence of the APOE e4 allele.

**Figure 1.**
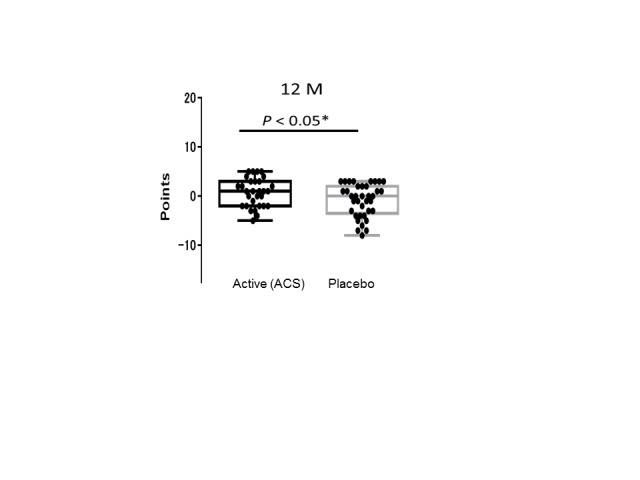
Longitudinal changes in WMS-LM2 scores. A box plot of the WMS-LM2 data in Table 2 for subjects in the active (n = 30) and placebo (n = 37) groups. Each black dot indicates the difference between the first and final test scores for one volunteer. Box shows the 25-75 percentile, and solid bar shows the median. (One subject in the active group could not complete the final WMS-LM2 test.)

## MATERIALS AND METHODS

### Subjects

Eighty-four healthy volunteers from 60-80 years of age were recruited in the Tokyo metropolitan area from April to August of 2014 [5]. Exclusion criteria were 1) a neuropsychiatric disorder or head injury; 2) a local lesion, such as a brain tumor or cerebral infarction, that could affect cognitive function; or 3) claustrophobia or a metal or electrical implant that would prevent obtaining MRI scans. All participants provided informed written consent. The participants visited the study site at the beginning of the study and 3, 6, and 12 months later. Participants were randomized into the ACS or placebo group. RCT-consistent assignment to the ACS or placebo group, determined by age and gender, was performed by Imepro Inc. (Tokyo, Japan). All participants and clinical and coordinating personnel were blinded to the group assignments for the duration of the study. The study was approved in 2012 by the Ethics Committees of the University of Tokyo and the National Center of Neurology and Psychiatry. The present report is an age-restricted sub-analysis from this healthy volunteer study, using data from 68 elderly volunteers who were at least 65 years old and completed the study (Table 1).

**Table 1:** Characteristics of participants in a 12-month RCT of ACS.

	Active group	Placebo group	*p value*
Age	71.3 (4.8)	71.8 (4.8)	0.70
Gender (M/F)	14/17	15/22	0.61
BMI	22.1 (2.1)	21.9 (2.3)	0.67
Education, years	14.7 (2.0)	14.3 (3.1)	0.52
APOE4^+^ / APOE4^-^	8/23	4/33	0.09

### Test formula

The test formula was a powder supplied by NH Foods Ltd. of Japan, derived from chicken meat and containing anserine and carnosine at a ratio of 3:1. The safety of this imidazole dipeptide supplement was verified in previous studies [23,24]. Participants in the active group received twice-daily doses of the imidazole dipeptide formula (500 mg/dose). The placebo formula contained an equivalent amount of essential amino acids, specifically L-lysine (43 mg/day) and L-histidine (150 mg/day), which were chosen because the enzymatic digestion of carnosine (250 mg/day) generates L-histidine (150 mg/day) and beta-alanine [5].

**Figure 2.**
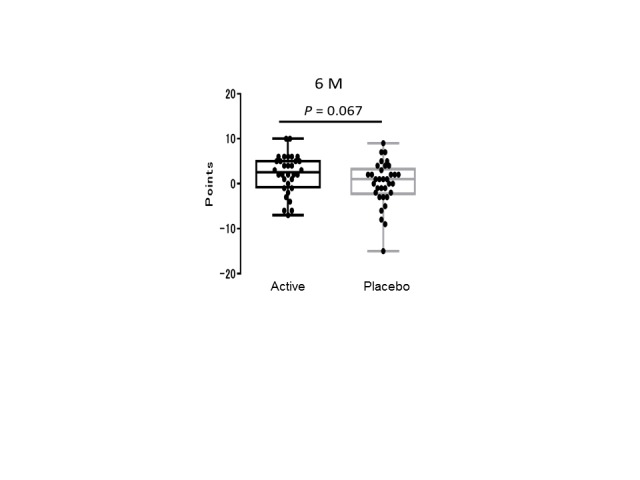
Longitudinal changes in WMS-LM2 scores at the mid-term test. A box plot of the change of WMS-LM2 story A data (the score of 6-month test - the score of the initial test) in the active and placebo groups. Each black dot indicates the difference between the first and final test scores for one volunteer. Box shows the 25-75 percentile, and solid bar shows the median.

### Inventory of anserine and carnosine in the normal diet

We calculated the amount of anserine and carnosine from the results of a dietary questionnaire, using a semi-quantitative method reported previously [5,25]. At follow-up, the participants filled out a self-administered questionnaire about the frequency of animal meat (chicken, pork, and beef) and fish meat (salmon, red-meat fish represented by tuna, white fish, blue-back fish represented by mackerel, and eel) in their diet over the previous 12 weeks. The representative fish in each category was chosen based on a national consumption survey. The average dietary intake was estimated based on the participants’ responses to the questionnaire and the concentrations of anserine and carnosine in the various animal and fish meats, obtained from our investigations and from Boldyrev et al. [6].

### Cognitive testing

We assessed the effect of ACS on cognitive function, mental status, and general health using the following cognitive-evaluation tools and self-reported questionnaires: 1) the Japanese version of the Wechsler Memory Scale-Revised Logical Memory immediate recall (WMS-LM1) and delayed recall (WMS-LM2) tests [5,11], 2) the Japanese version of a cognitive subscale of the Alzheimer’s Disease Assessment Scale (ADAScog) [5,26], and 3) the Mini Mental State Examination (MMSE) [5,27]. Mood and subjective states were assessed by the Japanese version of the Beck Depression Inventory (BDI) [5,28,29]. Mental and physical functional well-being was assessed by the Medical Outcomes Study, 36-item Short Form (SF-36) [5,30,31]. We calculated the Mental Health Component Summary (MCS) score and Physical Health Component Summary (PCS) score; higher scores indicated better function. Cognitive and psychological tests were performed under double-blind conditions.

### APOE genotyping

Genomic DNA was isolated from blood clots, and the APOE alleles were analyzed by PCR as previously described [32]. The subjects were subgrouped according to whether they carried the ε4 allele (APOE4^+^ subgroup) or not (APOE4^-^ subgroup), and within each subgroup, the psychological test scores and the changes in scores from baseline to the end of the trial were compared.

**Table 2:** Subjects grouped by APOE genotype.

	APOE4^+^ group			APOE4^-^ group		

	E4/E4	E4/E3	E4/E2	E3/E3	E3/E2	E2/E2
Active	1	7	0	23	0	0
Placebo	2	2	0	33	0	0

### MRI analysis

For MRI scans, a 3 T scanner (Siemens, MAGNETOM Verio 3.0T) with a 32-channel phased-array head coil was used. Headgear and earplugs were used to limit head motion and reduce scanner noise. Participants underwent MRI scans prior to beginning the trial and again after 12 months of supplementation. The 3D T1-weighted magnetization-prepared rapid gradient echo (MPRAGE) images were collected using the following parameters: TR = 1900 ms, TE = 2.52 ms, TI = 90 ms, flip angle = 9°, field of view (FoV) = 256 × 256 mm, acquisition matrix = 256 × 256, slice thickness = 1.0 mm, slice gap = 0 mm, slice number = 192 [33]. The 3D pulsed arterial spin labeling (pASL) perfusion images were collected by turbo gradient spin echo using the following parameters: TR = 5000 ms, TE = 38.8 ms, TI = 2350 ms, flip angle = 180°, FoV = 192 × 192 mm, acquisition matrix = 64 × 64, slice thickness = 3.0 mm, slice gap = 1.5 mm, slice number = 40, bolus duration = 700 ms. The 3D pASL data were calculated from the MRI data obtained at the start and end of the trial, and were analyzed using the statistical parametric mapping 12 (SPM12) system. The data were spatially normalized to MNI coordinates using the DARTEL method [34], and smoothed with a full-width parameter at a half-maximal resolution of 12 mm × 12 mm × 12 mm for 3D pASL as previously reported [12]. To compare changes in brain perfusion at the start and end of the 12-month trial, the data were spatially normalized to data from the 3D T1-weighted images, and the smoothed pASL data were subjected to two-way intra-subject analysis assessing the time and group interaction. We also obtained diffusion MRI and T2-weighted fluid-attenuated inversion-recovery (FLAIR) data for each participant. Diffusion tensor images were collected by diffusion-MRI with the following parameters: TR = 14100 ms, TE = 81 ms, flip angle = 90°, FoV = 224 × 224 mm, acquisition matrix = 114 × 114, slice thickness = 2.0 mm, slice number = 75, axes = 30, *b*-factor = 0, and 1000 s/mm^2^. T2-weighted FLAIR scans, used to confirm that there were no neurological or inflammatory disorders, were acquired with the following parameters: repetition time TR = 11000 ms, echo time TE = 94 ms, inversion time TI = 2800 ms, 20 axial slices, FoV = 198 × 220 mm. The diffusion tensors were calculated with the Diffusion Toolkit for the voxelwise statistical analysis [35]. Movements and eddy currents corrections were conducted using FSL eddy correct program for the diffusion-weighted images [36]. After correction, diffusion weighted images are skull-stripped with FSL BET program to exclude non-brain voxels from all analyses [37]. Fiber tract analysis and visualization were performed in the TrackVis program.

### Statistical analyses

Data were analyzed by a two-way repeated ANOVA or a two-tailed Student’s *t*-test. A *p* value less than 0.05 was considered significant. Unless stated otherwise, data were expressed as the mean ± SEM.

**Figure 3.**
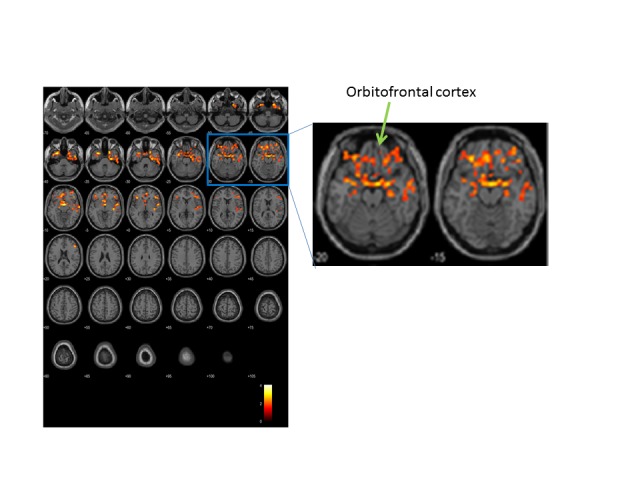
Longitudinal changes in brain perfusion. Brain blood flow was analyzed by ASL. Color indicates regions where changes in brain perfusion differed between the two groups (n = 31 active, 37 placebo). After repeated two-way ANOVA in SPM, the biggest difference was at (x, y, z) = (3, -1, -10) in Montreal Neurological Institute (MNI) coordinate, T=4.09; other two locations were (x, y, z) = (-30, 11, -37) and (24, 11, -43). SPM statistics showed significance between Active and Placebo (Active > Placebo). P(FWE-corr) < 0.001, KE=2857 voxels. Note that the brain locations of the preservation of blood flow by ACS included both sides of temporal lobes, orbitofrontal cortices, dorsolateral prefrontal cortices and anterior cingulate cortices.

**Table 3:** Anserine/carnosine intake from the diet^a^

	Food	Active Group Ave.±SEM	Placebo Group Ave.±SEM	*p* value
Anserine	Poultry (656mg/80g)	120.9±17.6	132.6±17.2	0.37
(mg/day)	Pork (18.4mg/80g)	5.1±0.7	4.9±0.6	0.66
	Beef (43mg/80g)	6.2±1.2	6.4±1.3	0.79
	Red meat Fish (304mg/80g)	77.1±10.9	71.6±8.9	0.5
	Blue back Fish (5.6mg/80g)	1.6±0.2	1.6±0.2	0.9
	White Fish (2.3mg/80g)	130.0±23.4	145.1±25.2	0.39
	Eel (0mg/80g)	0±0	0±0	N.D.
	Anserine(Total)	340.9±38.7	362.2±342.1	0.46
Carnosine	Poultry (184mg/80g)	33.9±4.9	37.2±4.8	0.37
(mg/day)	Pork (246mg/80g)	68.8±9.5	65.7±7.9	0.66
	Beef (209mg/80g)	30.0±6.0	31.1±6.1	0.79
	Red meat Fish (24mg/80g)	6.1±0.9	5.6±0.7	0.5
	Blue back Fish (152mg/80g)	43.5±5.9	44.0±5.2	0.9
	White Fish (0mg/80g)	0±0	0±0	N.D.
	Eel (336mg/80g)	7.3±0.8	7.6±0.7	0.65
	Carnosine(Total)	189.6±17.3	191.3±17.3	0.89

a Estimated intake anserine and carnosine was calculated from the results of a 7-item food frequency questionnaries filled out by each volunteer and the average amount of anserine and carnosine in each type of meat described by Boldyrev et al. (2013)

## RESULTS

### Subject data

At the end of the trial, 68 subjects had completed the study, with 12 months of ACS (31 subjects) or placebo (37 subjects) and a battery of tests at the start and end of the trial period. No obvious adverse events were observed. As shown in Table 1, the active and placebo groups did not differ significantly with respect to age, body mass index, sex ratio, or education. Of the subjects, 17.6% were APOE4*^+^*, and one subject in the active group and two in the placebo group were APOE4-homozygous (Table 2).

### Anserine/carnosine intake

The estimated dietary intake of anserine from animal and fish meat was 341 mg/day in the active group and 362 mg/day in the placebo group (Table 3). The estimated amount of carnosine from the diet was 190 mg/day in the active group, and 191 mg/day in the placebo group. These differences in intake in the normal diet were not significant. ACS provided 750 mg anserine and 250 mg carnosine per day. Thus, the active group ingested approximately three times more anserine/carnosine than the placebo group in this trial.

**Figure 4.**
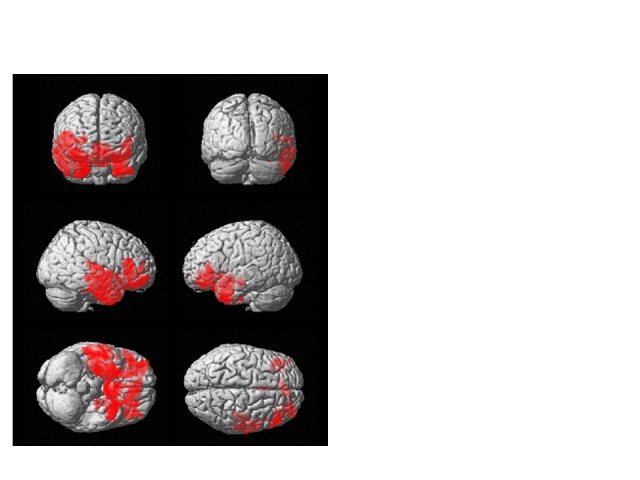
ACS preserves blood flow in the prefrontal brain of elderly people. Location of differential longitudinal changes in brain perfusion (red) in the active and placebo groups, on a brain montage from the SPM platform based on equivalent calculations to those in Fig. 3. In the active group, blood flow in these areas was elevated in the follow-up MRI scan.

**Figure 5.**
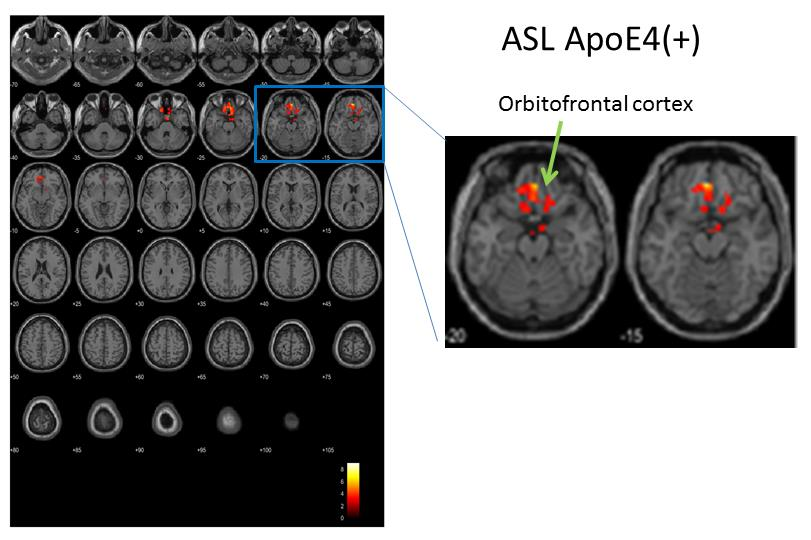
Longitudinal changes in brain perfusion in APOE4^+^ subjects, assessed by ASL. Color indicates brain regions with differences in longitudinal changes in brain perfusion between the active (n = 8) and placebo (n = 4) groups. After repeated two-way ANOVA in SPM, the biggest difference was at (x, y, z) = (-3, 44, -16), T=9.03. SPM statistics showed significance between Active and Placebo (Active > Placebo). P(FWE-corr) = 0.002, KE=378 voxels.

### Cognitive tests

Cognitive function was assessed using three neuropsychological tests (Table 4). Story B of the WMS-LM2 test was used to assess the delayed recall of verbal memory. Data were analyzed by two-way repeated ANOVA (Time [1^st^-test or 2^nd^-test] × Variant [ACS or placebo]). The interaction Time × Variant was significantly different between the ACS and placebo groups (F[1,65] = 4.2003, *p* = 0.0445; Fig. 1). In addition, at the mid-term test (6 months after the supplementation), we have observed a tendency (*p* = 0.067) of the cognitive improvement by ACS (Fig. 2). However, there was no difference between the active and placebo groups in the MMSE, the WMS-LM1 to assess the immediate recall of verbal memory, the ADAcog, the SF-36, or the BDI (F[1,65] < 1.0; *p* > 0.05).

### MRI analysis

**Figure 6.**
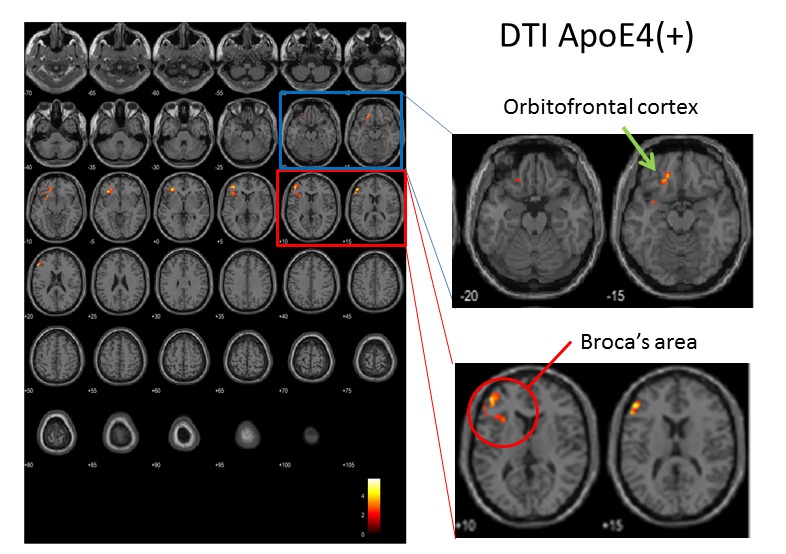
Longitudinal changes in FA (fraction anisotropy) values in APOE4^+^ subjects, assessed by DTI. Color indicates areas of the brain where FA values differed between the active (n = 8) and placebo (n = 4) groups. After repeated two-way ANOVA in SPM, the biggest difference was at (x, y, z) = (-32, 32, -4), T=5.75; other two locations were (x, y, z) = (-38, 44, 8) and (-50, 36, 18). SPM statistics showed significance between Active and Placebo (Active > Placebo). P(FWE-corr) = 0.003, KE=754 voxels.

**Figure 7.**
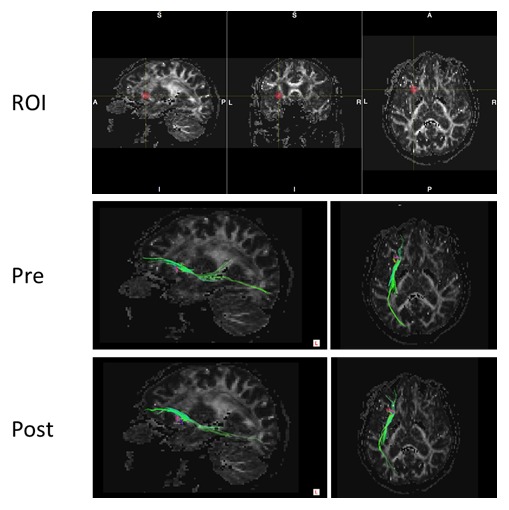
Tract-graph analysis of APOE4/E4 subject (#75) in the active group. Top panel shows a seed ROI (x, y, z) = (-30, 30, -4), Sphere Radius = 3.00 mm. Middle panel: a tract-graph using this spherical seed ROI at the start-up scan (Pre). Bottom panel: a tract-graph using this spherical seed ROI at the follow-up scan (Post). Note the longer tract (green) toward the frontal pole seemed to be stronger at the follow-up scan.

Longitudinal ACS-induced changes in brain perfusion were assessed by comparing the 3D pASL data between the ACS and placebo groups at the beginning and end of the trial. The analysis of whole-brain pASL data revealed a significant preservation of brain perfusion at the medial prefrontal cortex in the ACS group (*p* < 0.001; Fig. 3 and 4), whereas the inverse calculation did not reveal any significant findings. These changes were also analyzed within the APOE4^+^ and APOE4^-^ subgroups. We detected significant preservation of blood flow at the prefrontal areas in APOE4^+^ group (*p* = 0.002; Fig. 5), but not in APOE4^-^ group. We also compared the DTI data from diffusion MRIs between the ACS and placebo groups at the beginning and follow-up time points, and detected the significant difference between the two groups (*p* = 0.003; Fig. 6). Fig. 7 shows a tract-graph analysis of APOE4/E4 subject (#75) in the active group with a seed ROI at x=-30; y=30; z=-4, where we detected the difference between the two groups in the previous DTI analysis (Fig. 6). We observed that the longer tract (green) toward the frontal pole seemed to be stronger at the follow-up scan, in comparison with the tract-graph at the start-up scan.

## DISCUSSION

Here, we showed that ACS suppresses age-related memory decline and alteration in brain blood flow, in line with previous observations [4,5,10]. However, a new finding in the present study is that ACS has stronger benefits for elderly people bearing the risk allele APOE4, as revealed by both ASL and DTI data. Brain blood flow was preserved in prefrontal areas of the brain in APOE4 carriers after 12 months of ACS. Although considerable research has gone into determining whether DHA can benefit APOE4 carriers, the issue is still in debate [38,39]. ACS is another promising candidate for protecting against the detrimental effect of APOE4 molecules on brain function and structure.

Our research suggests that ACS may be able to suppress progressive memory decline, which often follows the onset of AD, in APOE4^+^ individuals, who are at a greater risk for AD [14,15]. There were not enough APOE4^+^ subjects in this study to demonstrate a significant difference in changes of verbal episodic memory score between APOE4^+^ subjects in the active and placebo groups, but we observed a marked trend toward improvement in the active group (n = 8; avg ± SD = 1.75 ± 2.76) compared to the placebo group (n = 4; -0.25 ± 5.25) in WMS-LM scores. We also observed a trend toward improvement in the MMSE test: the change in score was 0.75 ± 2.12 (n = 8) in the active group and -0.25 ± 1.89 (n = 4) in the placebo group. Among the APOE4-homozygous subjects, one subject was in the active group (Fig. 7); this 70-year-old man (#75) with a family history of dementia improved his verbal episodic memory score from 6 to 13. Two subjects in the placebo group were APOE4 homozygotes. One was a 76-year-old man (#42) whose verbal episodic memory score dropped severely, from 22 to 1, and he was diagnosed with mild cognitive impairment (MCI) after the 12-month trial. The other APOE4-homozygous subject in the placebo group (#38) was a 67-year-old man who maintained his verbal episodic memory score (15 at the first visit, and 17 at the 12-month follow-up visit).

**Table 4:** Changes in psychological test scores in the active and placebo groups.

	1^st^ test		Follow-up			Change	

	Active	Placebo	Active	Placebo	Active	Placebo	*p* value
WMS-LM-1^a^	13.3 (3.9)	15.0 (3.7)	14.3 (3.9)	14.3 (4.4)	0.93 (2.8)	-0.65 (3.6)	0.054
WMS-LM-2^a^	12.6 (4.0)	14.3 (3.9)	13.3 (3.9)	13.5 (4.4)	0.73 (2.9)	-0.84 (3.3)	0.044*
MMSE^b^	27.5 (1.7)	27.6 (1.9)	28.2 (2.4)	28.5 (1.7)	0.74 (2.0)	0.92 (1.9)	0.53
ADAS^b^	9.3 (5.5)	8.2 (4.2)	7.9 (4.6)	6.8 (4.4)	-1.4 (3.5)	-1.4 (3.3)	0.92
SF-36 PCS^b^	48.0 (7.7)	49.1 (6.6)	45.8 (10.2)	48.1 (5.8)	-2.2 (7.5)	-1.0 (6.1)	0.45
SF-36 MCS^b^	54.3 (4.7)	52.8 (5.8)	56.8 (6.9)^a^	52.4 (7.0)	2.3 (7.0)	-0.4 (7.4)	0.13
BDI^b^	8.2 (6.7)	6.8 (4.3)	8.0 (6.2)	7.2 (5.8)	-0.1 (3.7)	0.4 (4.5)	0.60

* Statistically significant.

a For the WMS-LM test combined story B, the 1^st^ test was performed at the second visit (3 months after beginning supplementation). Tests include 68 subjects except for the WMS-LM test, which analyzed only 67 subjects (n = 30 active and 37 placebo) because one subject in the active group did not complete the follow-up WMS-LM.

b The 1^st^ test was performed at the first visit; the follow-up test was conducted after 12 months of supplementation (n = 31 active, 37 placebo).

After our 12-month ACS RCT, we conducted a 12-week RCT of ACS in elderly patients with MCI. In the 12-week RCT, we observed that the MMSE scores were preserved and WMS-LM scores improved in MCI subjects carrying the APOE4 allele (n = 8 in the active group and n = 12 in the placebo group; unpublished observation, T.H.). Taken together, these findings suggest that ACS may protect against progressive cognitive decline in elderly individuals with the AD risk allele APOE4.

We also previously investigated the mechanism of the protective effect of ACS on cognitive function in a transgenic AD mouse model. We found that carnosine supplementation prevents memory deficits in the AD mouse, probably by preventing abnormalities in brain blood vessels [40]. Very recently, we obtained preliminary observations suggesting that anserine supplementation may prevent damage to brain microvascular pericytes (unpublished observation, J.K. & T.H. submitted separately). A series of studies by Zlokovic and colleagues indicate that pericyte degeneration contributes to the development of AD, especially in AD patients with the APOE4 allele [41,42]. We can speculate that once ingested, anserine and carnosine inhibit cellular damage to brain microvascular pericytes [15]. However, carnosine and anserine have several biochemical properties that could be involved in this effect, such as their anti-oxidant and anti-glycolytic activity and their ability to chelate metal ions [6,7,43], so further study is needed to determine the precise mechanisms. We are currently in the process of assessing whether ACS can protect against cognitive decline in a model mouse produced by crossing human APOE4/E4 knock-in [44] mice with AD (App/Psen double-transgenic) model mice [45] to generate an App/Psen double-transgenic mouse with a human ApoE4/E4 knock-in.

Normal aging is associated with diminished brain perfusion, which can be measured as cerebral blood flow by ASL [12]. Recent studies show that APOE e4 molecules accelerate the decline in brain blood flow at particular areas, including prefrontal regions [16,18]. In this study, we observed that the brain blood flow was preserved in elderly APOE e4 carriers who took ACS for a 12-month period, suggesting that ACS specifically protects against brain vascular abnormalities related to the APOE e4 molecule, such as those observed in brain microvascular pericytes [41,42]. In addition to ASL, other MRI modalities can also demonstrate the effect of the APOE e4 allele on brain aging, including structural MRI [46], functional MRI [47], and diffusion MRI [20]. In this report, an analysis of diffusion MRI data revealed that ACS protected against or improved white-matter abnormalities in APOE4+ subjects. Several well-designed clinical studies have shown that changes in white-matter microstructure in APOE4+ elderly individuals can be detected by analyzing the FA values after diffusion MRI [48-51]. In this study, the analysis of two MRI modalities, ASL and DTI, revealed that ACS protects against the putative detrimental effect of APOE e4 molecules on brain aging in healthy volunteers without dementia. In a separate 12 weeks-RCT, we have also observed preservation of cognitive functions by ACS in APOE4+ mild cognitive impairment patients (Active N=8, Placebo N=12; T.H. submitted separately).

### Limitations

Our study has various limitations. This study was based on a sub-analysis of ApoE4^+^ subjects within a larger study, and it had a relatively small sample size. Larger studies are still needed to verify the effect of ACS in elderly people carrying the APOE4 allele. In addition, it is possible that alterations in blood flow in the brain occur more rapidly in APOE4^+^ than in APOE4^-^ individuals, so longer-term investigations should be performed.

### Concluding Remarks

This is the first RCT to demonstrate the effect of ACS in healthy APOE4^+^ volunteers. The results indicate that ACS may protect against age-related structural changes in the brain in elderly people with the APOE4 allele, and thus prevent Alzheimer’s Disease if started before the MCI stage. Furthermore, changes seen on MRI scans, particularly ASL and DTI, are potential biomarkers for assessing these benefits, especially in people carrying the APOE4 allele.
